# Person-Centered Care Practice, Patient Safety Competence, and Patient Safety Nursing Activities of Nurses Working in Geriatric Hospitals

**DOI:** 10.3390/ijerph18105169

**Published:** 2021-05-13

**Authors:** Ayoung Huh, Juh Hyun Shin

**Affiliations:** 1 Graduate School of Clinical Health Science, Ewha Woman’s University, Seoul 03760, Korea; 2 College of Nursing, Science & Ewha Research Institute of Nursing Science, Ewha Womans University, Seoul 120750, Korea; juhshin@ewha.ac.kr

**Keywords:** person-centered care, patient safety competence, patient safety nursing activities, geriatric hospital

## Abstract

Geriatric nursing activities are closely related to patient safety; therefore, nurses’ efforts to improve patient safety in geriatric hospitals are important. In the present study, we investigated the relationships between person-centered care practice, patient safety competence, and patient safety during nursing activities in geriatric hospitals. We used the following tools to investigate the factors affecting patient safety during nursing activities: (a) the Korean version of the Person-Centered Care Assessment Tool (P-CAT), (b) the Patient Safety Competence Assessment Tool for Nurses, and (c) the Patient Safety Nursing Activities Assessment Tool for geriatric nurses. The questionnaire survey was completed by 186 geriatric nurses in 12 geriatric hospitals from 1 August to 31 August 2018. We analyzed the survey data using a *t*-test, one-way ANOVA, Pearson’s correlation coefficient, and multiple regression. We identified patient safety skills (β = 0.417, *p* < 0.001) and age (β = 0.209, *p* = 0.035) as key factors that influence patient safety during nursing activities. Therefore, to improve the quality of patient safety during nursing activities conducted by geriatric nurses, it is necessary to develop strategies to improve patient safety skills and expand the pool of competent nurses with clinical experience.

## 1. Introduction

### 1.1. Necessity of Research

The number of long-term-care geriatric nursing hospitals (hereinafter referred to as “geriatric hospitals”) in Korea has doubled over the past decade, from 777 in 2009 to 1577 in 2019 [1]. Along with their occupancy rate, the proportion of long-term-care insurance coverage for elderly beneficiaries relative to the general population is steadily increasing (from 7.0% in 2015 to 9.6% in 2019) [2]. With the rapid increase in the number of geriatric hospitals, accessibility to healthcare services for elderly patients in need of medical and nursing care has also improved. However, the provider-centered healthcare approach has resulted in impaired quality of services, including patient safety issues [3]. According to the Patient Safety Statistics Yearbook, 18.4% of the total number of patient safety incidents in 2019 (2198 out of 11,953 cases) occurred in geriatric hospitals. Safety incidents were most frequently caused by falls (*n* = 1684, 31.8%), followed by medication errors, infections, and medical equipment errors [4]. Nursing activities are closely related to patient safety; for example, nurses assist with medication, infection control, and fall prevention. Thus, nurses’ efforts to improve patient safety are crucial in geriatric hospitals [5,6].

Patient safety nursing activities are nursing activities undertaken to ensure patient safety in hospitals. Most importantly, these activities involve the prediction and prevention of safety problems that are likely to occur during the treatment process [7]. Geriatric hospital patients are prone to safety incidents such as falls, fractures, lacerations, and bedsores due to impaired sensory and cognitive functions caused by various age-related chronic and degenerative diseases such as high blood pressure, diabetes, dementia, Parkinson’s disease, cardiovascular disease, stroke, hearing and visual impairment, and senile depression [8]. When safety incidents occur, elderly patients with multiple comorbidities are more likely to experience worse outcomes in terms of mortality, complications, and quality of life compared to younger patients [9]. For this reason, elderly patients’ safety incidents not only directly affect their lives but also degrade the quality of healthcare services due to prolonged hospitalizations and increased inpatient costs. In this context, patient safety nursing activities of geriatric nurses are crucial [10].

Nurses’ patient safety competence influences patient safety nursing activities [7,11,12]. Patient safety competence refers to the attitude, skills, and knowledge that healthcare workers should have to protect patients from unnecessary risks and hazards [13]. Geriatric hospitals in Korea function as an intermediate station between acute-care hospitals and nursing homes, inevitably accommodating patients with a wide spectrum of chronic or acute diseases and thus requiring highly competent nurses for geriatric care [8]. However, compared to the importance attached, the competency levels of geriatric nurses have been found to be rather low. Therefore, it is necessary to assess and improve the nursing competency levels of geriatric nurses to improve patient safety [14].

The mean length of a hospital stay in a geriatric hospital in Korea is as long as 2.5 years due to the lack of alternative services to replace inpatient care of the elderly [8]. In Korea, geriatric hospitals also function as social facilities where elderly patients with therapeutic and nursing needs can stay on a long-term basis. It has been suggested that geriatric nurses need to implement holistic, person-centered care that meets the patients’ physical, psychological, and social needs [3]. Person-centered care refers to the awareness and practice of showing respectful attitudes toward patients’ individual abilities and values and assisting them in maintaining their autonomy, self-esteem, and independence to fulfill their psychological needs [15]. Holistic, person-centered care enhances physical, social, and emotional aspects of the patients’ life through the formation of a sound therapeutic rapport between patients and nurses [16]. Additionally, patient-centered care improves patient safety by reducing the risks associated with impairment while carrying out activities of daily living. Patient-centered care also reduces fall risks, restraint use, pressure ulcers, behavioral symptoms, and psychotropic medication use in dementia patients [17,18,19,20]. Furthermore, person-centered care promotes mutual support and cooperation by facilitating a patient–nurse relationship of mutual trust and support [21], thereby improving the quality of nursing service and patient safety [3,22,23].

This study analyzes factors influencing person-centered care practice, patient safety competence, and patient safety nursing activities of geriatric nurses to enhance elderly patients’ overall quality of life considering physical, social, and emotional factors. We also aimed to provide fundamental data for developing plans to improve patients’ safety in geriatric hospitals.

### 1.2. Purpose of the Study

This study was conducted to analyze factors influencing person-centered care practice, patient safety competence, and patient safety nursing activities of geriatric nurses. Specifically, we sought to analyze and determine the following: (a) general characteristics (demographic, educational, and work experience related information) of geriatric nurses; (b) levels of person-centered care practice of geriatric nurses along with nurses’ patient safety competence, and patient safety nursing activities; (c) differences in the levels of person-centered care practice, patient safety competence, and patient safety nursing activities according to nurses’ general characteristics; (d) correlations between person-centered patient care practices, patient safety competence, and patient safety nursing activities of geriatric nurses; and (e) factors influencing geriatric nurses’ patient safety nursing activities.

## 2. Methods

### 2.1. Design and Participants

This study adopted a descriptive survey design. We performed convenience sampling in 12 geriatric hospitals located in Provinces S and G. The selection criteria were nurses working in geriatric hospitals who understood the purpose and procedure of this study and voluntarily agreed to participate in the study. Considering the high turnover rate of geriatric nurses [24], nurses with at least 6 months of experience were included in the study. The sample size was set at 190, by adding a 10% expected dropout rate in 172 participants; the required sample size was calculated at the significance level of 0.05, medium effect size of 0.15, and power of 0.95, using the G*power 3.1.9.2 program (HHU, Dusseldorf, Nordrhein-westfalen, Germany). After excluding four questionnaires with missing responses, 186 questionnaires were used for the analysis.

### 2.2. Research Tools

General characteristics: Based on the findings of previous studies on geriatric hospitals [5,25], the following items were examined: demographic characteristics, education level, clinical experience (years of total experience and experience in the current workplace), and experience with patient safety (education, work, and incidents).

Person-centered care: To assess person-centered care practice, we used the person-centered care assessment tool (P-CAT) developed by Edvardsson et al. [26]. The P-CAT was translated and adapted for the Korean population (K-P-CAT) and the reliability and validity of the K-P-CAT were tested by Tak et al. [15]. The P-CAT is a 13-item questionnaire designed to measure the extent of personalized care. The K-P-CAT consists of two subscales consisting of items on person-centeredness (seven items) and organizational and environmental support (six items), of which five items are reverse-coded. Each item is rated on a 5-point Likert-type scale ranging from 1 (completely disagree) to 5 (completely agree), where a higher score indicates a higher degree of person-centered care. Sentence comprehension of the K-P-CAT was confirmed by Sagong and Lee [3], who performed a preliminary survey with five nurses. The Cronbach’s α (reliability coefficient) of the P-CAT was 0.84 at the time of its development [26], and that of the K-P-CAT in the study by Tak et al. [15] was 0.86. In the current study, Cronbach’s α was 0.78.

Patient safety competence: We used the questionnaire developed by Lee and Jang, which they described as “questionnaires to measure baccalaureate nursing students’ patient safety competencies” [27]. The questionnaire measures the patient safety competence that nursing students should attain with graduation. This 41-item scale consists of three subscales: knowledge, skills, and attitude. Each item is rated on a 5-point Likert-type scale, with a total score ranging from 41 to 205, with high scores indicating high competency. Jang [13] verified the content validity of the questionnaire by testing it on nurses with clinical experience, and its construct validity and reliability were verified accordingly. The Cronbach’s α of the tool was 0.91 at the time of development [27], 0.95 in the study by Jang [13], and 0.96 in the current study.

Patient safety nursing activities: The Patient Safety Nursing Activity Assessment Tool was developed by Park et al. [28] by extracting patient safety nursing tasks from the assessment tool used to evaluate healthcare providers, as developed by the Korea Health Industry Development Institute in 2007. The version revised by Moon and Yoon was used in the current study [5]. The 68-item questionnaire was grouped into nine subdimensions: fall prevention (12 items), infection control (9 items), personal identification (3 items), patient education (5 items), communication (4 items), medication (14 items), blood transfusion (16 items), firefighting (4 items), and equipment check (1 item). Each item is rated on a 5-point Likert-type scale ranging from 1 (I never perform it) to 5 (I always perform it), where a higher score indicates a higher frequency of the corresponding patient safety nursing activity. The Cronbach’s α values in the study of Park et al. [28], the study of Moon and Yoon [5], and this study were 0.97, 0.98, and 0.97, respectively.

### 2.3. Data Collection

Data were collected from 1 to 31 August 2018, following the approval of the Institutional Review Board (IRB). We visited the nursing department of each of the 12 hospitals and presented the study and survey to the nurses. After explaining the purpose of the study and the questionnaire completion method, we obtained written consent from each participant and administered the questionnaire. The participants were instructed to carefully read and complete the questionnaire, which took about 20 min to complete. To ensure anonymity, each participant was asked to seal the signed consent form and the completed questionnaire and submit them personally to the researchers. Of the 190 questionnaires distributed, 186 were included for analysis after excluding four questionnaires with incomplete answers.

### 2.4. Data Analysis

The collected data were analyzed using the SPSS 23.0 (IBM Corporation, Armonk, NY, USA). The general characteristics of the participants were analyzed using frequency analysis. The levels of person-centered care practice, patient safety competence, and patient safety nursing activities of the participants were analyzed using descriptive statistics (mean and standard deviation). The differences in these factors according to the participants’ general characteristics were analyzed using independent sample t-tests and a one-way ANOVA, and a post hoc analysis was performed using Scheffé’s test. The correlations between the participants’ person-centered care practice, patient safety competence, and patient safety nursing activities were analyzed using Pearson’s correlation coefficients. Factors influencing patient safety nursing activities were analyzed using multiple regression analysis.

## 3. Results

### 3.1. General Characteristics of the Participants

A significant majority of the nurses (*n* = 184, 98.9%) were women, and a large majority (*n* = 145, 78.0%) were married. The mean age of the sample (*n* = 186) was 43.61 years. Nursing college graduates with a 3-year diploma constituted the largest group (*n* = 122, 65.6%). The mean length of clinical career was 12.75 years (±102.29) in total and 2.66 years (±31.90) in the current workplace (geriatric hospital). In terms of patient safety, 167 participants (89.8%) received a training course, 146 participants (78.5%) had no working experience in the patient safety area, and 153 participants (82.3%) had experience with patient safety incidents. Drawing on the research finding that 5-year turnover rates are significantly related to turnover intention among geriatric nurses [20], we categorized the clinical experience in 5-year increments (see Table 1).

### 3.2. Levels of Person-Centered Care Practice, Patient Safety Competence, and Patient Safety Nursing Activities of the Participants

The participants’ mean score for person-centered care practice was 3.27 ± 0.43. In patient safety competence, the overall mean score was 3.92 ± 0.44, and those of its subdimensions, namely, attitude, skills, and knowledge, were 4.33 ± 0.45, 3.84 ± 0.52, and 3.27 ± 0.76, respectively. The mean score for patient safety nursing activities was 4.52 ± 0.42 (see Table 2). The highest mean score was in the patient safety area of fall prevention (4.64 ± 0.42), whereas the lowest was in communication (4.34 ± 0.68).

### 3.3. Differences in Person-Centered Care Practice, Patient Safety Competence, and Patient Safety Nursing Activities according to the Participants’ General Characteristics

No statistically significant differences were observed in person-centered care according to the general characteristics. Regarding patient safety competence, statistically significant differences were observed in age (*F* = 3.040, *p* = 0.030), education level (*F* = 6.552, *p* = 0.002), total length of clinical career (*F* = 3.158, *p* = 0.026), patient safety education (*t* = 2.271, *p* = 0.024), and patient-safety-related work experience (*t* = 4.577, *p* < 0.001). Post hoc comparisons using Scheffé’s test revealed significant differences in the age groups of 30 s and >50 s. Higher patient safety competence scores were observed in the older age group, the higher-education-level group, the groups with experience of patient safety education, and the groups with experience in patient-safety-related work. In terms of patient safety nursing activities, statistically significant differences were observed in patient-safety-related work experience (*t* = 2.384, *p* = 0.019). Moreover, patient safety competence scores were high in the group with higher patient-safety-related work experience (see Table 3).

### 3.4. Correlations between Person-Centered Care Practice, Patient Safety Competence, and Patient Safety Nursing Activities

Patient safety nursing activities were significantly correlated with all three patient safety competence subdimensions (attitude −*r* = 0.204, *p* = 0.005; knowledge −*r* = 0.292, *p* < 0.001; skill −*r* = 0.451, *p* < 0.001). Moreover, patient safety knowledge was significantly correlated to patient safety attitude (*r* = 0.214, *p* = 0.003), skill (*r* = 0.648, *p* < 0.001), and person-centered care (*r* = 0.159, *p* = 0.030; see Table 4).

### 3.5. Factors Influencing Patient Safety Nursing Activities

To determine the factors that influence geriatric nurses’ patient safety nursing activities, we performed multiple regression analysis using the person-centered care practice, the three subdimensions of patient safety competence (attitude, skills, and knowledge), and general characteristics (demographics, clinical career, and patient safety experience) of the participants as independent variables. As a result of checking the multicollinearity problem, the tolerance value was less than 1.0, and the variance inflation factor (VIF) was under the standard value of 10; thus, the model was considered to be free from the multicollinearity problem. The analysis revealed that the participants’ patient safety skills (β = 0.417, *p* < 0.001) and age (β = 0.209, *p* = 0.035) were found to have significant effects on patient safety nursing activities, with the explanatory power of the model calculated at 27.4% (*R*^2^ = 0.274). The regression equation was statistically significant (*F* = 4.048, *p* < 0.001; see Table 5).

## 4. Discussion

This study was conducted to determine the correlations between person-centered care practice, patient safety competence, and patient safety nursing activities in geriatric hospitals and to identify the factors affecting patient safety nursing activities.

The mean score for patient safety nursing activities was 4.52, which was higher than that obtained in an earlier study with geriatric nurses using the same measurement tool [7] and in another study with tertiary hospital nurses using a different tool, but with a similar structure and items [12]. Patient safety is of particular importance in geriatric hospitals because these hospitals cater to a wide spectrum of patient conditions. Patients in geriatric hospitals are susceptible to safety accidents related to conditions such as arthritis; cardiovascular disease; and those requiring protective body gear or diapers, sedatives and sleeping pills, vasodilators, brain function enhancers, and antibiotics [6]. This allows the assumption that nurses working in geriatric hospitals are more sensitized to patient safety than those working in general hospitals.

Among the subdimensions of patient safety nursing activities, fall prevention showed the highest score, which is consistent with a similar study [7]. This may be due to geriatric nurses’ awareness that falls are a major cause of patient safety incidents, leading to complications from fractures, prolonged hospital stays, and even death [29]; therefore, proactive fall-related safety measures are in place [29].

Infection control activities ranked the second highest. This is inconsistent with an earlier study that showed geriatric nurses scored lower in their infection control practices than perceived by them [30]. The most common infections in geriatric hospitals are urinary tract infections, followed by respiratory tract infections and skin infections, with the incidence of nosocomial infections estimated at 2.8–32.7% or 1.80–13.5 cases per 1000 days of hospital stay [31]. Because a geriatric hospital is a long-term healthcare facility for the elderly with impaired immune function, the importance of infection control cannot be underestimated. The high degree of infection control activities among geriatric nurses seems to reflect their awareness of the high risk of nosocomial infection among geriatric patients.

Among the subdimensions of patient safety nursing activities, communication scored the lowest, which is similar to previous results that showed communication in high-risk hospital environments tends to be unilateral, negative, or insufficient [32]. This finding also reflects geriatric nurses’ beliefs about communication as an unimportant area of patient safety nursing activities. However, organizational communication has been reported to be an important factor in promoting patient safety nursing activities of geriatric nurses [7], and improving the effectiveness of communication is one of the National Patient Safety Goals set by the Joint Commission on Accreditation of Healthcare Organizations [33]. In this context, there is a need to develop strategies to raise awareness and improve communication skills among medical staff.

The overall mean score for the level of patient safety competence was 3.92 points, which is lower than the results obtained in other studies with tertiary hospital nurses using the same assessment tool [12,13]. The result of this study is consistent with the previous result [14], which found that nursing competence levels were lower compared to how geriatric nurses perceive its importance. This score highlights the need to promote geriatric nurses’ patient safety competence. Among the three subdimensions, patient safety attitude and patient safety knowledge scored the highest and lowest marks with 4.33 and 3.27 points, respectively, which is consistent with the report that geriatric nurses perceive the importance of nursing competence as high but their competence levels fall short of their perception [14]. Considering the report that patient safety knowledge influences patient safety nursing activities [12], it is necessary to develop systematic educational and training courses for geriatric nurses to build and improve their patient safety knowledge.

The mean score for the degree of person-centered care practice was 3.27; this score was higher than that calculated in a study with geriatric nurses using the same assessment tool [3] but lower than that calculated in a study with geriatric-care-facility employees [15]. This difference between geriatric hospitals and care facilities supports the report [34] that services in geriatric hospitals are more focused on healthcare than on well-being compared to services in geriatric care facilities in terms of policy efforts related to holistic care of inpatients and employees’ practice of personalized patient care. In an international comparison, studies conducted in Spain and Sweden [35,36] reported higher levels of person-centered care practice, presumably due to a longer history of long-term care schemes and person-centered care along with interest in geriatric patients’ quality of life. This highlights the need to develop policy strategies to enhance person-centered care practices tailored to Korea’s geriatric hospital environment and to improve geriatric nurses’ perceptions and awareness of holistic and person-centered care.

Our analysis of the differences in the level of patient safety competence depending on the participants’ general characteristics revealed statistically significant differences in age, education level, experience of patient safety education, and experience of working in the patient safety area. The positive correlation between age and patient safety competence supports the result [14] that nursing skills and competence improve with increasing age and clinical experience. In terms of education level, nurses with postgraduate and higher educational accomplishments scored the highest in patient safety competence, which may be attributable to the fact that postgraduate and specialized nursing programs enhance problem-solving abilities across nursing-related subareas and procedures. Therefore, it is necessary to encourage geriatric nurses to learn and continue building personal competence.

A statistically significant positive correlation was observed between patient safety work experience and patient safety nursing activities; moreover, the groups that scored high in the patient safety work experience also scored high in patient safety competence. This is similar to the report [12] that patient safety nursing activities vary depending on whether a nurse has participated in patient-safety-related activities. The experience of managing and supervising patient-safety incidents and educating others about patient safety provides nurses with an opportunity to accumulate knowledge of patient safety, acquire patient safety competence, and engage in patient safety nursing activities.

A statistically significant positive correlation was also observed between patient-centered care and patient safety knowledge. This result supports the report [21] that the practice of person-centered care is facilitated by employees’ specialized knowledge and that clinical knowledge and practice should be constantly updated. Moreover, all three subdimensions of patient safety competence (attitude, skills, and knowledge) were found to have significant positive correlations with patient safety nursing activities. That is, a geriatric nurse with a higher score of patient safety competence can better carry out patient safety nursing activities, which in turn is associated with higher scores in patient safety attitude, patient safety skills, and patient safety knowledge, similar to the results of the aforementioned study [12] with tertiary hospital nurses. To promote geriatric nurses’ patient safety nursing activities, it is necessary to develop strategies to improve nurses’ patient safety competence.

Analysis of the factors influencing patient safety nursing activities revealed that nurses with higher patient safety skills (one of the three subdimensions of patient safety competence) performed patient safety nursing activities more frequently. Nurses who have acquired good patient safety skills can systematically assess and observe patients’ conditions to prevent potential problems [13]. Therefore, it is necessary to recognize the importance of nurses’ patient safety skills at the organizational level and to develop education programs using various media such as simulation to improve patient safety skills.

Nurses’ age was found to have a positive effect on patient safety nursing activities, presumably due to the fact that younger nurses have less experience than older nurses, which is associated with weaker decision-making authority and work autonomy, as reported by a study with tertiary hospital nurses [37]. This result also aligns with the reports [38,39] that young nurses in geriatric hospitals are more challenged than their counterparts working in acute care hospitals. Therefore, geriatric hospitals need to improve workplace environments to secure experienced nurses and set up strategies to reduce the turnover rate of nurses by promoting nursing skills tailored to geriatric patient care.

In this study, person-centered care practice was found to have no significant effect on patient safety nursing activities, thus failing to support the result that the former is a key factor for the latter. However, previous studies [17,18,19,20] have reported that person-centered care practice in geriatric care facilities impacts the performance of patient safety activities of employees, thus improving patient safety; reducing the incidence of falls, fractures, and abrasions; and lowering the rates of pressure ulcers and restraint and psychotropic medication use in dementia patients. Further studies are necessary to analyze the relationship between person-centered care practice and patient safety nursing activities in geriatric hospitals. In addition, we found that geriatric nurses’ perception of the need for person-centered care does not lead to the actual performance of patient safety nursing activities [38]. This finding may be associated with the fact that geriatric nurses spend a large amount of time on paperwork for documents requested by assessment agencies and frequent death processing, which leaves them less time for direct nursing hours [39]. This highlights the need to explore ways to help geriatric nurses better focus on direct nursing activities by ensuring sufficient nursing staff and improving the work environment.

In this study, the age and patient safety skills of geriatric nurses were identified as variables that have positive effects on patient safety nursing activities. Therefore, in order to enhance the quality of healthcare services in geriatric hospitals, it is important to secure older nurses with long and diverse clinical experience and improve their patient safety skills. A limitation of this study is that the small sample size consisting of nurses recruited from 12 geriatric hospitals extracted through convenience sampling makes the generalizability of its results difficult. This study has the following limitations: (a) the data collection relied on self-report questionnaires, and (b) the study is biased toward nurses in geriatric hospitals at which participants are currently employed. Participants may have also felt fatigued because it took 20 min on average to complete 122 questions. Additionally, this study was conducted before the COVID-19 pandemic; therefore, newly revised policies were not studied. Further research should study these new policies regarding placement of infection-control staff, strengthening education of infection control and tightening hospital standards, configuring assessment items, and regularly monitoring to prevent infectious disease [40,41]. More research with extensive samples reflecting current healthcare environments, including the COVID-19 pandemic, is required in the future.

## 5. Conclusions

This descriptive survey study was conducted to investigate the relationships between person-centered care practice, patient safety competence, and patient safety nursing activities of geriatric hospital nurses and to analyze the factors affecting patient safety nursing activities. The analysis identified that patient safety skills and age positively affected patient safety nursing activities.

The significance of this study lies in the fact that it is the first study in Korea to analyze the levels of geriatric nurses’ person-centered care practice, patient safety competence, and patient safety nursing activities. Extending this study, in-depth research on person-centered care practice and patient safety may be conducted with a larger sample from more regions.

Considering the lower score for person-centered care practice compared with long-term geriatric care facilities, it is necessary to adopt a more holistic approach to geriatric nursing care that encompasses the patients’ physical, emotional, and social needs. Additionally, in view of the low scores for patient safety knowledge compared with patient safety attitude and patient safety skills and the fact that nurses with postgraduate level scored the highest marks in patient safety competence, it is proposed to establish policy measures for continuing education of geriatric nurses and mandatory placement of nurses specialized in geriatric care.

## Figures and Tables

**Table 1:** General characteristics of the participants (*N* = 186).

Variables	Categories	*n* (%)
Gender	Male	2 (1.1)
	Female	184 (98.9)
Age (Years)	≤30	34 (18.3)
	31–40	41 (22.0)
	41–50	51 (27.4)
	>50	60 (32.3)
	Mean ± SD	43.61 ± 10.98
Marital status	Unmarried	39 (21.0)
	Married	145 (78.0)
	Others	2 (1.1)
Education level (Degree)	3-Year diploma	122 (65.6)
	Bachelor	54 (29.0)
	Master	10 (5.4)
Total length of career (Years)	≤5	41 (22.0)
	5<~≤10	44 (23.7)
	10<~≤15	34 (18.3)
	>15	67 (36.0)
	Mean ± SD	12.9 ± 102.29
Career in current long-term care hospital (Years)	≤1	47 (25.3)
	1<~≤2	44 (23.7)
	2<~≤3	41 (22.0)
	>3	54 (29.0)
	Mean ± SD	2.8 ± 31.90
Experience of patient safety education	Yes	167 (89.8)
	No	19 (10.2)
Experience of patient safety work	Yes	40 (21.5)
	No	146 (78.5)
Experience of patient safety accident	Yes	153 (82.3)
	No	33 (17.7)

**Table 2:** Degree of person-centered care, patient safety competence, and patient safety nursing activities (*N* = 186).

Variables		Min–Max Values	M ± SD
Person-centered care		2.23–4.77	3.27 ± 0.43
Patient safety competence	Patient safety attitude	3.00–5.00	4.33 ± 0.45
	Patient safety skill	2.71–5.00	3.84 ± 0.52
	Patient safety knowledge	1.00–5.00	3.27 ± 0.76
	Total	2.85–4.98	3.92 ± 0.44
Patient safety nursing activities	Fall down	3.25–5.00	4.64 ± 0.42
	Infection	2.89–5.00	4.62 ± 0.48
	Maintenance facilities	3.00–5.00	4.55 ± 0.65
	Personal identification	2.33–5.00	4.54 ± 0.56
	Blood transfusion	2.69–5.00	4.54 ± 0.47
	Medication	2.71–5.00	4.45 ± 0.52
	Fire fighting	1.75–5.00	4.41 ± 0.71
	Patient education	2.20–5.00	4.39 ± 0.56
	Communication	2.00–5.00	4.34 ± 0.68
	Total	2.74–5.00	4.52 ± 0.42

**Table 3:** Differences in person-centered care, patient safety competence, and patient safety nursing activities by general characteristics (*N* = 186).

		Person-Centered Care			Patient Safety Competence				Patient Safety Nursing Activities		
Variables	Categories	M ± SD	t/F	*p*	M ± SD	t/F	*p*	Scheffé	M ± SD	t/F	*p*
Age (year)	≤30 ^a^	3.32 ± 0.36	2.118	0.100	3.83 ± 0.39	3.040	0.030 *	b < d	4.45 ± 0.42	1.400	0.244
	31–40 ^b^	3.14 ± 0.36			3.79 ± 0.39				4.49 ± 0.48		
	41–50 ^c^	3.24 ± 0.50			3.97 ± 0.50				4.49 ± 0.43		
	>50 ^d^	3.34 ± 0.43			4.02 ± 0.42				4.61 ± 0.36		
Marital status	Single/etc.	3.24 ± 0.35	−0.367	0.714	3.87 ± 0.39	−0.827	0.410		4.52 ± 0.42	0.010	0.992
	Married	3.27 ± 0.45			3.93 ± 0.45				4.52 ± 0.42		
Education level	3 year diploma ^e^	3.24 ± 0.45	0.912	0.404	3.93 ± 0.44	6.552	0.002 **	e,f < g	4.51 ± 0.44	0.311	0.733
	Bachelor’s degree ^f^	3.30 ± 0.39			3.83 ± 0.39				4.56 ± 0.37		
	Master’s or higher ^g^	3.41 ± 0.29			4.36 ± 0.43				4.48 ± 0.50		
Total length of career (year)	≤5	3.34 ± 0.42	1.553	0.202	3.84 ± 0.41	1.988	0.117		4.52 ± 0.37	0.075	0.973
	5<~≤10	3.26 ± 0.41			3.86 ± 0.42				4.51 ± 0.45		
	10<~≤15	3.14 ± 0.43			3.90 ± 0.44				4.50 ± 0.48		
	>15	3.30 ± 0.44			4.02 ± 0.46				4.54 ± 0.40		
Career in current geriatric hospital (year)	≤1	3.31 ± 0.45	0.636	0.593	3.88 ± 0.48	0.471	0.703		4.43 ± 0.50	1.235	0.298
	1<~≤2	3.19 ± 0.42			3.88 ± 0.42				4.57 ± 0.37		
	2<~≤3	3.27 ± 0.44			3.95 ± 0.38				4.51 ± 0.42		
	>3	3.28 ± 0.40			3.97 ± 0.46				4.57 ± 0.38		
Experience of patient safety education	Yes	3.26 ± 0.43	−0.062	0.951	3.95 ± 0.44	2.271	0.024 *		4.52 ± 0.42	0.196	0.845
	No	3.27 ± 0.38			3.71 ± 0.32				4.50 ± 0.41		
Experience of patient safety work	Yes	3.34 ± 0.50	1.144	0.258	4.19 ± 0.45	4.577	<0.001 ***		4.63 ± 0.31	2.384	0.019 *
	No	3.24 ± 0.40			3.85 ± 0.41				4.49 ± 0.44		
Experience of patient safety accident	Yes	3.26 ± 0.44	−0.245	0.807	3.93 ± 0.41	0.513	0.611		4.51 ± 0.42	−0.682	0.496
	No	3.28 ± 0.37			3.88 ± 0.55				4.57 ± 0.43		

^a^: age group of 30 or under; ^b^: age group from 31 to 40; ^c^: age group from 41 to 50; ^d^: age group of over 50; ^e^: group of 3 year diploma; ^f^: group of Bachelor’s degree; ^g^: group of Master’ degree or more * *p* < 0.05, ** *p* < 0.01, *** *p* < 0.001.

**Table 4:** Correlations between person-centered care, patient safety competence, and patient safety nursing activities. (*N* = 186).

Variables	Person-Centered Care	Patient Safety Attitude	Patient Safety Skill	Patient Safety Knowledge
	r (*p*)	r (*p*)	r (*p*)	r (*p*)
Patient safety attitude	−0.086 (0.244)			
Patient safety skill	0.143 (0.051)	0.415 (<0.001) ***		
Patient safety knowledge	0.159 (0.030) *	0.214 (0.003) **	0.648 (<0.001) ***	
Patient safety nursing activities	0.091 (0.220)	0.204 (0.005) **	0.451 (<0.001) ***	0.292 (<0.001) ***

* *p* < 0.05, ** *p* < 0.01, *** *p* < 0.001.

**Table 5:** Factors influencing patient safety nursing activities (*N* = 186).

Variables	Patient Safety Nursing Activities					
	B	SE	β	t	*p*	VIF
Person-centered care	0.051	0.10.	0.036	0.498	0.619	1.089
Patient safety attitude	0.003	0.091	0.003	0.032	0.975	1.335
Patient safety skill	0.338	0.084	0.417	4.042	<0.001 ***	2.206
Patient safety knowledge	0.004	0.054	0.007	0.080	0.937	1.841
Gender	0.007	0.276	0.002	0.024	0.981	1.071
Age	0.008	0.004	0.209	2.129	0.035 *	1.991
Education level	−0.014	0.054	−020	−0.267	0.790	1.184
Total length of career	−0.005	0.002	−0.171	−2.148	0.033	1.308
Career in current geriatric hospital	0.009	0.013	0.060	0.721	0.472	1.426
Experience of patient safety education	−0.028	0.015	−0.149	−1.879	0.062	1.299
Experience of patient safety work	0.061	0.078	0.062	0.783	0.435	1.301
Experience of patient safety accident	−0.052	0.084	−0.046	−0.618	0.537	1.155

* *p* < 0.05, *** *p* < 0.001. *F* = 4.048 ***, *R*^2^ = 0.274, adj *R*^2^ = 0.206.

## Data Availability

Not avaliable.
